# Engaging Communities With a Simple Tool to Help Increase Immunization Coverage

**DOI:** 10.9745/GHSP-D-14-00180

**Published:** 2015-03-02

**Authors:** Manish Jain, Gunjan Taneja, Ruhul Amin, Robert Steinglass, Michael Favin

**Affiliations:** a Maternal and Child Integrated Program (MCHIP)/India. Now with India Health Action Trust/Technical Support Unit, Uttar Pradesh, India; b MCHIP/India. Now with IPE Global, Jharkhand, India; c MCHIP/Timor-Leste. Now with LuxDev, Vientiane, Lao People's Democratic Republic; d MCHIP. Now with the Maternal and Child Survival Project and John Snow, Inc, Washington DC,, USA; e MCHIP. Now with the Maternal and Child Survival Project and The Manoff Group, Washington DC, USA

## Abstract

The level of vaccination coverage in a given community depends on both service factors and the degree to which the public understands and trusts the immunization process. This article describes an approach that aims to raise awareness and boost demand. Developed in India, the “My Village Is My Home” (MVMH) tool, known as *Uma Imunizasaun* (UI) in Timor-Leste, is a poster-sized material used by volunteers and health workers to record the births and vaccination dates of every infant in a community. Introduction of the tool in 5 districts of India (April 2012 to March 2013) and in 7 initial villages in Timor-Leste (beginning in January 2012) allowed community leaders, volunteers, and health workers to monitor the vaccination status of every young child and guided reminder and motivational visits. In 3 districts of India, we analyzed data on vaccination coverage and timeliness before and during use of the tool; in 2 other districts, analysis was based only on data for new births during use of the tool. In Timor-Leste, we compared UI data from the 3 villages with the most complete data with data for the same villages from the vaccination registers from the previous year. In both countries, we also obtained qualitative data about perceptions of the tool through interviews with health workers and community members. Assessments in both countries found evidence suggesting improved vaccination timeliness and coverage. In India, pilot communities had 80% or higher coverage of identified and eligible children for all vaccines. In comparison, overall coverage in the respective districts during the same time period was much lower, at 49% to 69%. In Timor-Leste, both the number of infants identified and immunized rose substantially with use of the tool compared with the previous year (236 vs. 155, respectively, identified as targets; 185 vs. 147, respectively, received Penta 3). Although data challenges limit firm conclusions, the experiences in both countries suggest that “My Village Is My Home” is a promising tool that has the potential to broaden program coverage by marshalling both community residents and health workers to track individual children's vaccinations. Three states in India have adopted the tool, and Timor-Leste had also planned to scale-up the initiative.

## Abstract (precis)

Use of a simple, publicly placed tool that monitors vaccination coverage in a community has potential to broaden program coverage by keeping both the community and the health system informed about every infant's vaccination status.

## BACKGROUND

Low vaccination coverage often reflects services that are not sufficiently accessible, convenient, reliable, or friendly. It may also be related to a lack of public understanding or trust in vaccination and/or vaccination services. In addition, various sociocultural factors commonly correlate with the likelihood of families getting their children immunized.^1^^,^^2^

Most interventions aimed at raising vaccination coverage focus on improving services or on informing and motivating families. This article describes an approach, piloted in India and Timor-Leste, which aims to improve demand and also has the potential to affect services: use of a tool that enables community members or community-based health workers to list all infants in the community and monitor their individual vaccinations. The tool is called “My Village Is My Home” (MVMH) in India and *Uma Imunizasaun* (immunization house or UI) in Timor-Leste.

**Figure 1.**
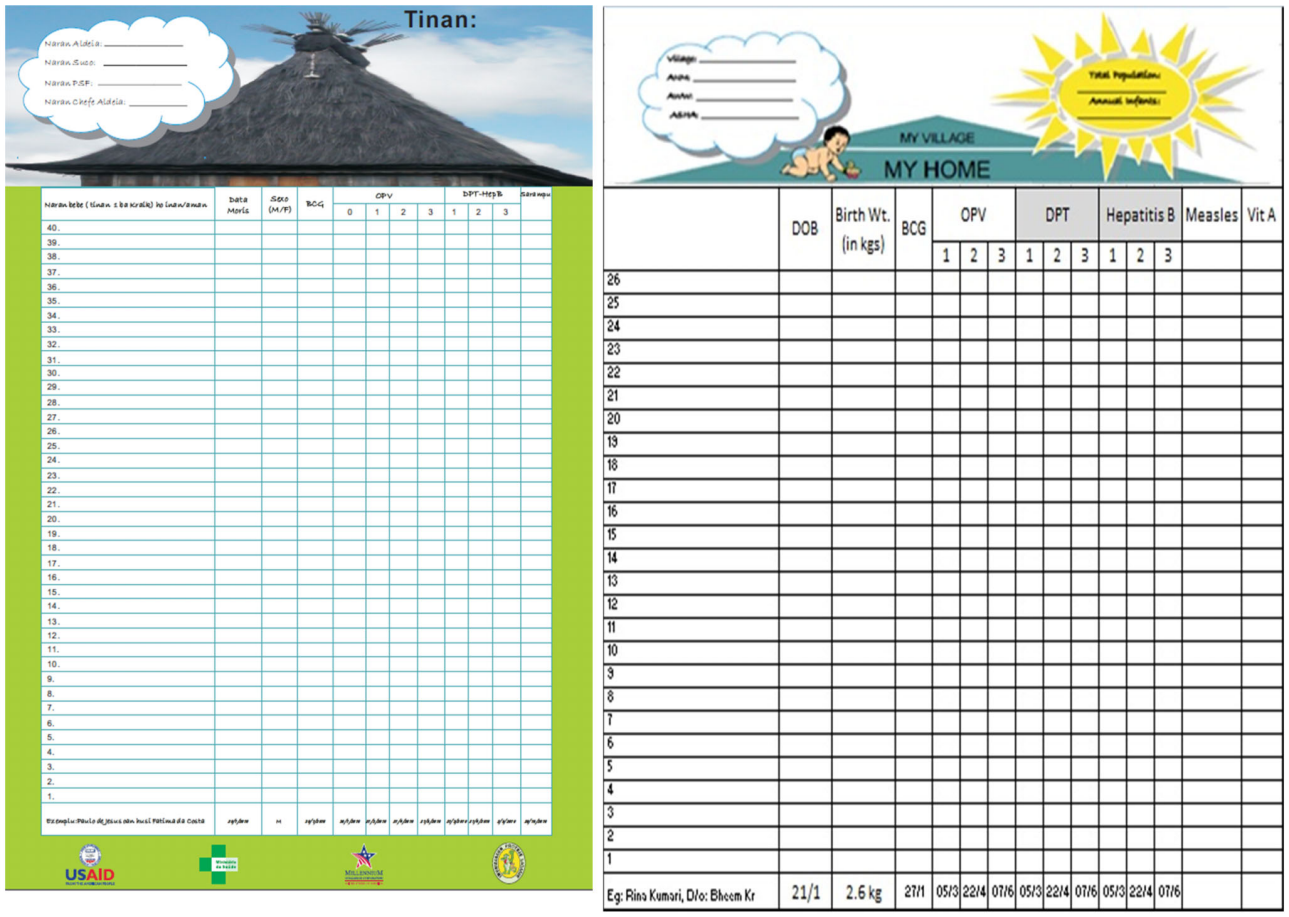
Community-Based Tool to Monitor Immunization Coverage: *Uma Imunizasaun* in Timor-Leste (left) and “My Village Is My Home” in India (right)

The MVMH/UI tool is a large, poster-sized record on which every infant in a community has his or her own row, with spaces for the child's name, date of birth, and dates of each vaccination (Figure 1). Yearly, local volunteers or health workers do a rapid census to fill in the names and birth dates of all infants, starting with the oldest in the bottom row and moving upward. Infants born or moving into the community during the year are added on the next open line above. A roof covers the listings, illustrating the idea that each vaccination of each child fills in a brick or board that strengthens the entire house, which is equivalent to adding protection for the entire community from vaccine-preventable diseases.

The “My Village Is My Home” tool allows a community to track all vaccinations for every infant in the population.

The tool was developed in India in 2003 to take advantage of the 700,000 Anganwadi Workers (AWWs) (community-based workers) who conducted annual censuses of infants and women. Posted in a public place such as a community center or local government office, the large MVMH/UI is meant to create social pressure to keep one's children up-to-date on vaccinations. Ideally, use of the tool should inform and motivate caregivers, local leaders, and volunteers, as well as professional health staff, to have more infants vaccinated, and sooner. (In some settings, posting such personal information in a public place might raise concerns about privacy issues; however, we heard no such concerns in the 2 pilot settings.)

## COUNTRY PROGRAM DESCRIPTIONS

### India

In India, most vaccinations are given through outreach services delivered at Anganwadi Centers (AWCs), which are government day care centers. The Maternal and Child Health Integrated Program (MCHIP), with support from the United States Agency for International Development (USAID), undertook the MVMH initiative from April 2012 to March 2013 in 28 AWCs in Deoghar and Jamtara districts of Jharkhand and between August 2012 and July 2013 in 15 AWCs in Banda, Gonda, and Varanasi districts in Uttar Pradesh. Project and government staff trained several cadres of health workers to use the MVMH tool, including Auxiliary Nurse-Midwives (ANMs), who deliver immunization services at these outreach session sites; Accredited Social Health Activists (ASHAs), who are the community volunteers from the health department; and AWWs, the community workers from the social welfare department, at all 43 AWCs. Training focused on listing infants' names in birth order and updating their row when they received each vaccination. Data entry into the MVMH tool rested primarily with the ANMs, while mobilization of children for services rested with the ASHAs and AWWs.

**Figure:**
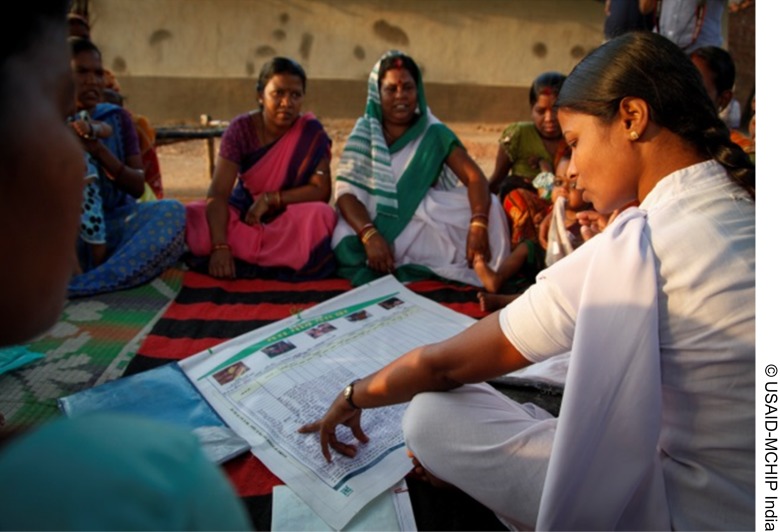
A health worker in India uses the “My Village Is My Home” tool at a vaccination session.

Recording new vaccinations was relatively easy in India, because under the Universal Immunization Program there are fixed days designated for immunization services at all outreach sites. The tool served as a displayed “due list” that showed the specific children and vaccinations due at the next session. It guided the local team to alert those families just before the vaccination day.

### Timor-Leste

In Timor-Leste, the UI tool was an initiative of the *Imunizsaun Proteje Labarik* (Immunization Protects Children or IPL) project under MCHIP, with funding from the Millennium Challenge Corporation. IPL developed a Timorese version of the tool, a simple manual for community volunteers, and a curriculum for training local elected leaders and *Promotores Saude Familiar* (PSF) (family health promoters, or community health volunteers). Following a 2-day national workshop in September 2011 for national immunization partners, project and local Ministry of Health (MOH) staff trained the community implementers in late 2011 in 7 pilot *sucos* (villages), which introduced the tool in early 2012. Later in 2012, IPL collaborated with the NGO *Clinic Café Timor*, which introduced the tool into another 26 *sucos*. Prior to IPL's closure in December 2013, the project and the MOH introduced the tool in an additional 21 low-performing subdistricts.

Local volunteers were trained to list all infants' names and birth dates and to record the date of each vaccination. They learned the ideal vaccination schedule and the required intervals between successive doses of polio and pentavalent vaccines (Table 1). The volunteers learned how to tell when a child was eligible or late for a particular vaccination and were encouraged to motivate caregivers of such infants to take them to community health centers, health posts, monthly integrated health outreach services, or mobile clinics to receive those vaccinations.

**Table 1.** Timor-Leste Immunization Schedule

**Vaccines**	**When To Give**	**Do NOT Give**
OPV 0	From birth to 2 weeks of age	After the first 2 weeks of age
BCG	As soon as possible after birth	After reaching 12 months of age
OPV 1/ Penta 1^a^	As soon as possible after 6 weeks of age	Before 6 weeks of age or after reaching 2 years of age
OPV 2/ Penta 2	As soon as possible after 10 weeks of age*And* at least 4 weeks after dose 1	Before 10 weeks of age or after reaching 2 years of age
OPV 3/ Penta 3	As soon as possible after 14 weeks of age*And* at least 4 weeks after dose 2	Before 14 weeks of age or after reaching 2 years of age
Measles	As soon as possible after reaching 9 months (39 weeks) of age	Before 9 months (39 weeks) of age

Abbreviations: BCG, bacille Calmette-Guérin; OPV, oral polio vaccine; Penta, pentavalent.

a Pentavalent vaccine in Timor-Leste protects against diphtheria, pertussis, tetanus, hepatitis B, and *Haemophilus influenzae* type b (Hib).

In Timor-Leste, the local volunteers completed a small UI at the *aldeia* (hamlet) level, and the information from all 5 to 10 *aldeias* in 1 *suco* was consolidated periodically on a large UI at the *suco* level. PSF, together with the elected hamlet or village chief, initially filled in the names and birth dates of the infants. Since vaccinations were not offered in most *aldeias,* the local volunteers met at the *suco* council office with the vaccinator from the subdistrict community health center, monthly if possible. IPL offered lunch and a small transport allowance for persons attending these meetings, at which the *aldeia* and *suco* tools were updated and discussed. If a PSF could not convince a family to have its children vaccinated, he or she could ask the vaccinator to visit that family. Unfortunately, too many children lacked their child health booklets for those to be the basis for local updating.

## ASSESSMENT METHODS

Neither project planned a priori to evaluate use of the tool. However, given the impressions of all involved that the tools were working well, both projects carried out assessments. The Institutional Review Board of John Snow, Inc. granted exemptions from review for the analyses for both India and Timor-Leste.

In India, data from all session sites were entered into an Excel spreadsheet, and coverage and timeliness of vaccination were analyzed. At project start in Uttar Pradesh, the team collected vaccination data before implementing the intervention (for the period from April 1, 2011 to July 31, 2012). The intervention was initiated on August 1, 2012 and completed on July 30, 2013. Thus, pre-intervention data were available for 16 months and intervention data for 10 months.

The data from Jharkhand, however, included only the follow-up component without any pre-intervention data. In addition, the tool in Jharkhand was used to record only new births that occurred during the study period from April 2012–March 2013. Thus, the analysis was based on the number of children who were eligible for the different vaccine doses per their birth dates.

In addition to the quantitative data, we also obtained qualitative data in India on health workers' and community members' perceptions about the usefulness of the tool through informal conversations.

In Timor-Leste, IPL first monitored use of the UI tool by interviewing parents, local leaders and volunteers, and local health staff in May 2012. Several months before the project ended, the IPL monitoring officer asked IPL district staff to take digital photos of the *suco* tools and health facility registers of a comparable, non-intervention *suco* for comparison. Major data problems soon became clear. Some information on the tools was illegible or did not make sense. Both numerator and population data from the subdistrict facilities were incomplete and/or unreliable. It was decided to compare UI data with data from the vaccination registers for the same *suco*s from the previous year. The monitoring officer entered and cleaned the data in Excel from the 3 *suco*s with the most complete data and carried out various analyses to gauge timeliness and coverage.

## RESULTS

### India

All 455 children who were born in the Jharkhand communities during the study period (April 2012–March 2013) were eligible for BCG (bacille Calmette-Guérin), and 437 of them received the vaccine. Similarly, 445 with birth dates on or before February 15, 2013 were eligible for the first dose of diphtheria, tetanus, and pertussis vaccine (DTP 1), the first dose of oral polio vaccine (OPV 1), and the first dose of hepatitis B (Hep B 1); 410 infants with birth dates on or before January 15, 2013 were eligible for the second doses of the same vaccines (DTP 2, OPV 2, and Hep B 2); and 377 infants born on or before December 15, 2012 were eligible for the third doses (DTP 3, OPV 3, and Hep B 3). By March 2013, 128 infants had completed the age of at least 9 months and were evaluated for measles vaccination. Overall, the coverage rates for eligible children in the study areas in Jharkhand were consistently high, with coverage rates for all the vaccines at more than 80% (Table 2), and the unimmunized rates at just 1.9%.

**Table 2.** Vaccination Coverage Rates During Use of the “My Home Is My Village” Tool, Jharkhand, India, April 2012–March 2013

**Antigens**	**No. of Children Eligible**	**No. of Children Vaccinated**	**Vaccination Coverage**
BCG	455	437	96.0%
OPV 1	445	417	93.7%
OPV 2	410	361	88.0%
OPV 3	377	306	81.2%
DTP 1	445	421	94.6%
DTP 2	410	367	89.5%
DTP 3	377	313	83.0%
Hep B 1	445	418	93.9%
Hep B 2	410	362	88.3%
Hep B 3	377	311	82.5%
Measles	128	104	81.3%

Abbreviations: BCG, bacille Calmette-Guérin; DTP, diphtheria, tetanus, pertussis; Hep B, hepatitis B; OPV, oral polio vaccine.

The MVMH districts had been poor performing, and surveyed full immunization coverage during this period was 48.6% in Deoghar and 68.6% in Jamtara (Annual Health Survey 2011–12 data). The surveyed coverage put BCG–measles dropout rates in both districts at close to 25%, while it was 15% in the study area. Another important impact in the study areas was on the timeliness of vaccination. Only 2.3% of children for OPV 1 and 3.0% for DTP 1 were immunized before 42 days, and the intervals from OPV 2 to OPV 3 and DTP 2 to DTP 3 were less than the minimal 28 days in only 3.5% and 3.4% of beneficiaries, respectively.

In Uttar Pradesh, increased coverage rates were noted after the intervention for all vaccines except measles, and the rate of unimmunized children also decreased, from 12.6% to 6.7% (Table 3). During the intervention period, 9.6% of children were immunized before the recommended age for DTP 1, 5.0% for DTP 3, and 9.6% for measles vaccination.

**Table 3.** Vaccination Coverage Rates Before (April 2011–July 2012) and During (August 2012–July 2013) Introduction of the “My Home Is My Village” Tool, Uttar Pradesh, India

**Vaccine**	**Pre-Intervention Cohort (N = 565)**			**Intervention Cohort^a^ (N = 868)**		
	**No. of Children Eligible**	**No. of Children Vaccinated**	**Vaccination Coverage**	**No. of Children Eligible**	**No. of Children Vaccinated**	**Vaccination Coverage**
BCG	565	465	82.3%	868	768	88.5%
OPV 0	565	306	54.2%	868	510	58.8%
DTP 1	506	423	83.6%	868	747	86.1%
DTP 3	444	306	68.9%	848	611	72.1%
Measles	280	200	71.4%	642	430	67.0%

Abbreviations: BCG, bacille Calmette-Guérin; DTP, diphtheria, tetanus, pertussis; OPV, oral polio vaccine.

a Includes children from the pre-intervention cohort plus new children born during the intervention period.

In Uttar Pradesh, the rate of unimmunized children decreased with use of the tool, from 12.6% to 6.7%.

Almost all health workers and community members interviewed during the study period were satisfied with the tool and felt that it had greatly contributed to improving the community's overall awareness of the need and importance of the immunization program.

### Timor-Leste

Table 4 contains illustrative data from the 3 *suco*s in Timor-Leste. The 2011 (pre-intervention) data for these areas suggested good immunization rates (over 90% for all antigens), so it is difficult to show a major impact of UI on vaccination coverage. During introduction of the UI tool, coverage seemingly dropped for some antigens. For example, the data suggest that coverage for Penta 3 decreased from 91% to 76%, and for measles, from 92% to 53%. However, the number of infants identified in 2012 (N = 236) was much higher than in 2011 (N = 155). This inconsistency suggests that the 2011 denominator used was too low, resulting in an inflated coverage rate for 2011. Even for 2012, the project staff believed that a small percentage of infants did not make it onto the UIs because they lived far from the community center or because their families traveled outside the community at times. (Much of the population lives in isolated mountain communities; 22.7% of 1-year-olds had no vaccinations in the 2009/10 Demographic and Health Survey.)

**Table 4.** Coverage and Timeliness of Vaccination Before (2011) and During (2012) Introduction of the UI Tool, 3 Villages of Timor-Leste

****	**BCG**		**Penta 1**		**Penta 3**		**Measles**	
****	**Before (N = 155)**	**During (N = 236)**	**Before (N = 155)**	**During (N = 236)**	**Before (N = 155)**	**During (N = 236)**	**Before (N = 155)**	**During (N = 236)**
Eligible to receive antigen, n (%)	155 (100)	236 (100)	155 (100)	236 (100)	155 (100)	236 (100)	147 (95)	194 (82)
Received antigen, n (%)	154 (99)	232 (98)	152 (98)	218 (92)	147 (95)	184 (78)	137 (93)	110 (57)
Age at vaccination, mean, days for BCG, weeks for all others	35	24	12	9	24	21	44	41
Vaccinated within recommended age range,^a^ n (%)	57 (37)	104 (45)	62 (41)	111 (51)	43 (29)	50 (27)	104 (76)	89 (81)
Vaccinated too early, n (%)	-	-	17 (11)	46 (21)	4 (3)	6 (3)	19 (14)	18 (16)

Abbreviations: BCG, bacille Calmette-Guérin; Penta, pentavalent; UI, *Uma Imunizasaun*.

a For BCG, data refer to those vaccinated between 0–13 days.

To do their intended job, vaccines must be given when they will work for each child, as laid out in the starting ages and intervals in national vaccination schedules. The data in Timor-Leste indicate a serious problem of vaccinations given to children who are too young (not yet eligible) and therefore who consequently may have gained no or reduced benefit from the administered antigen. This problem is particularly serious for Penta 1 and measles vaccinations, for which 21% and 16% of children, respectively, were vaccinated too early during use of the UI tool (Table 4). In contrast, before introduction of the UI tool, 11% and 14% of children were vaccinated too early for Penta 1 and measles, respectively.

In addition, the mean age for administration of OPV 0 was 27 days before introduction of the UI tool and 18 days during use of the UI tool (data not shown), indicating that many doses of OPV 0 were given to children over 14 days of age, the official cutoff age. OPV 0 vaccinations given after 2 weeks of age may reduce the antigenic response to the OPV 1 vaccination, depending on the interval between OPV 0 and OPV 1.

Almost no children were vaccinated for measles without having received BCG or DTP earlier (4% before use of the UI tool vs. 1% during use of the tool, data not shown). This is difficult to interpret but implies that health workers were not excessively missing opportunities to immunize. In addition, most children received all 3 Penta doses, although the data seem to suggest a small drop during use of the UI tool (94% before vs. 83% during, data not shown).

Overall, the prevalence of incomplete, inconsistent, and unexpected data reflected endemic problems with data in the health system.

The May 2012 interviews with parents, local leaders, volunteers, and local health staff uncovered that the purpose of the tool and processes were well understood and that most respondents felt very positively about the tool. Volunteers wanted support for transport and per diem. Implementation occurred as planned in 5 of the original 7 *sucos* but not in 2 due to inactive PSF.

**Figure:**
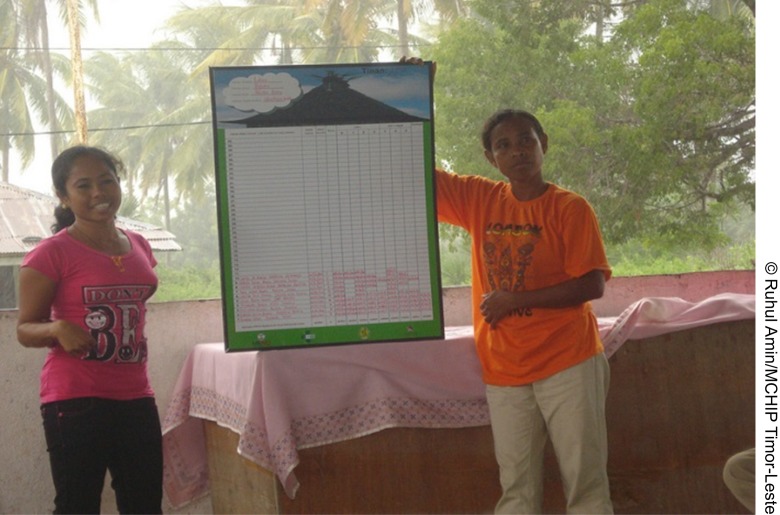
Health workers in Timor-Leste explain the *Uma Imunizasaun* tool to caregivers.

The UI tool was one of a half dozen project interventions assessed in a program review just before the project closed in late 2013. When interviewed during the project's final assessment, two-thirds of MOH and national partner staff viewed the UI tool as one of IPL's most effective activities. Local informants noted that the UI allowed them to know whether individual and all children in a community were up-to-date on vaccinations. It enabled them to mobilize their communities and to motivate parents of children who had missed vaccinations. One participant said: “It is easy to identify children under 1 year and follow-up for the next immunization. We also understand the immunization and interval dates.”

Most community leaders, PSF, vaccinators, and health managers hoped that the government would continue the activity and expand it to all *suco*s. However, several noted that this would require continued support for meals, fuel, and travel expenses, and that the MOH should be responsible for these costs. A few vaccinators and health managers commented that although it was a good tool, it increased their workload.

## DISCUSSION

Recent studies and reviews have associated increased community participation in immunization with improvements in vaccination coverage.^3^^–^^5^ Use of the MVMH/UI tool in 2 very diverse settings made health workers and families more aware of the current vaccination status of individual children and motivated them to improve coverage. Although lack of reliable comparative data did not allow a valid measurement of impact on coverage, positive impacts are very likely. In Timor-Leste, coverage from the previous year was based on a denominator that was likely much lower than the real target population, and the number of infants tracked and immunized rose substantially with use of the tool (155 vs. 236, respectively, identified as “targets”; 147 vs. 185, respectively, received Penta 3). It appears that the apparent drop in coverage with use of the UI tool in Timor-Leste is attributable to the fact that previously only the most accessible infants were in the system and those harder to reach were left out. In India, coverage in pilot communities increased to much higher than overall coverage in their respective districts after MVMH was introduced.

Use of the tool improved awareness of vaccination status and motivated health workers and families to increase coverage.

Quantifying the tool's impact on coverage is also complicated by the fact that the projects in both India and Timor-Leste implemented multiple initiatives to improve services as well as community understanding and demand. The fact that the efforts of both projects were associated with coverage increases at the district level is likely attributable to this comprehensive approach.

Data from both countries show improvements in vaccinations not being given late, which brings great benefits to children, particularly in settings where vaccine-preventable diseases are common. However, there may be a common problem of children receiving antigens *before* they are eligible by age. This was a potentially important and unexpected finding that deserves study in other settings. Children who are vaccinated too early may not develop a sufficient antigen response to the vaccine, leaving them more vulnerable to disease.

Children receiving vaccines before they are eligible by age was a finding that deserves further study.

Qualitative feedback indicates that use of the tool helped increase awareness of vaccination and a sense of shared responsibility between health services and communities. Everyone involved—local health workers, volunteers, and caregivers—felt very positively about the tool and its impact on vaccination in the communities, although some vaccinators in Timor-Leste noted that updating the tool entailed time and extra work for them.

Cost-effectiveness was not analyzed, although it appears that the costs are modest and depend on many local factors. Regular updating of the tool may be relatively easy, as in India, or more challenging, as in Timor-Leste, depending on such factors as prevalence and completeness of home-based vaccination records, feasibility of communication via cell phone, and availability of community-based health workers and volunteers.

**Figure 2.**
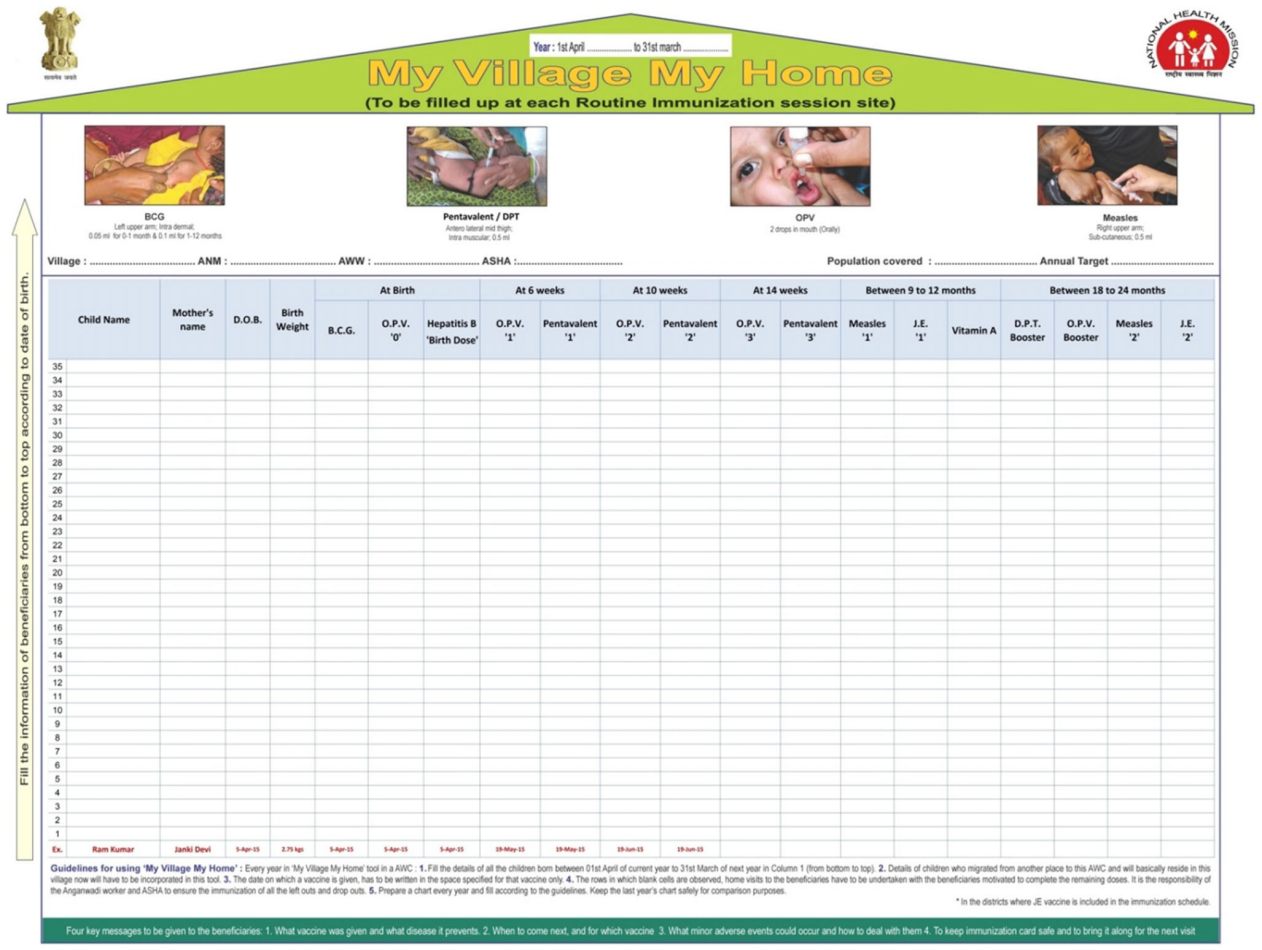
Revised MVMH Tool in India

In India, the tool design has been modified to: (a) incorporate birth doses recently added to the immunization schedule; (b) order the columns by the ideal age for each antigen rather than grouping multiple doses of the same antigen together; (c) serve as a reminder of the correct site, dose, and route of administration as well as of the 4 key messages for caregivers; and (d) give clear instructions in the local language on how to use the tool (Figure 2).

The MVMH tool has been available in the Indian setting since 2003, and the current intervention suggests that the tool is beneficial and easy to adapt. It has already been adopted by the state governments of Jharkhand and Uttar Pradesh, with approval to use the tool across all 77,000 AWCs in the 2 states. In addition, Haryana state has adopted MVMH for implementation. In 2014, the Alliance for Immunization partnered with numerous civil society organizations in Bihar, Jharkhand, Rajasthan, and Uttar Pradesh to use MVMH.^6^

Several state governments and civil society organizations in India have adopted the MVMH tool.

At the end of the IPL project in Timor-Leste, the MOH stated its intention of scaling-up use of the UI tool under a Gavi-funded health systems strengthening support project. In addition, other projects in the country were considering adapting the tool for communities to monitor recommended practices during pregnancy and postpartum.

In Timor-Leste, the MOH is intending to scale-up use of the UI tool, and other projects are considering adapting it for pregnancy/postpartum tracking.

While this tool shows promise, it is important to keep in mind that its scale-up for wider application, as with any scale-up, calls for attention to many implementation components, including the accompanying and critical community mobilization activities as well as local adaptation requirements.

## CONCLUSION

“My Village Is My Home” is a promising tool that can strengthen community participation in immunization. It has the potential to increase demand for immunization within health services and among the public, increase identification of young children requiring immunization, improve timeliness of vaccination, and boost coverage. Further trials and evaluation of its ability to improve vaccination coverage as well as community participation are merited.

## Notes

**Peer Reviewed**
